# 4-(Dimethyl­amino)benzaldehyde 4-ethyl­thio­semicarbazone

**DOI:** 10.1107/S1600536808037148

**Published:** 2008-11-13

**Authors:** Abdussalam Salhin, Norfarhah Abdul Razak, I. A. Rahman

**Affiliations:** a School of Chemical Sciences, Universiti Sains Malaysia, 11800 USM, Penang, Malaysia

## Abstract

The title thio­semicarbazone derivative, C_12_H_18_N_4_S, features intra­molecular N—H⋯N and C—H⋯S hydrogen bonds which generate *S*(5) ring motifs. The dihedral angle between the benzene ring and the thio­urea unit is 6.30 (6)° indicating planarity in the mol­ecule. Inter­molecular N—H⋯S hydrogen bonds generate dimers with an *R*_2_^2^(8) ring motif. The methyl group of the *N*-ethyl residue is disordered and was refined with site occupancies of 0.521 (5) and 0.479 (5).

## Related literature

For details of hydrogen-bond motifs, see: Bernstein *et al.* (1995 ▶). For related structures and applications see: Beraldo *et al.* (2001 ▶); Kayed *et al.* (2008 ▶); Valdes-Martinez *et al.* (1990 ▶).
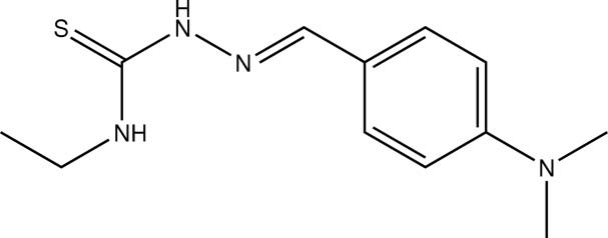


## Experimental

### 

#### Crystal data

C_12_H_18_N_4_S
*M* *_r_* = 250.36
Monoclinic, 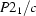

*a* = 9.3687 (2) Å
*b* = 14.1872 (3) Å
*c* = 10.2318 (2) Å
β = 96.699 (1)°
*V* = 1350.68 (5) Å^3^
*Z* = 4
Mo *K*α radiation
μ = 0.23 mm^−1^
*T* = 100 (1) K
0.59 × 0.36 × 0.22 mm

#### Data collection

Bruker SMART APEXII CCD area-detector diffractometer
Absorption correction: multi-scan (**SADABS**; Bruker, 2005 ▶) *T*_min_ = 0.879, *T*_max_ = 0.953
30229 measured reflections
6825 independent reflections
5566 reflections with *I* > 2σ(*I*)
*R*_int_ = 0.036

#### Refinement

*R*[*F* ^2^ > 2σ(*F* ^2^)] = 0.041
*wR*(*F* ^2^) = 0.119
*S* = 1.04
6825 reflections
176 parameters
H atoms treated by a mixture of independent and constrained refinement
Δρ_max_ = 0.74 e Å^−3^
Δρ_min_ = −0.39 e Å^−3^

### 

Data collection: *APEX2* (Bruker, 2005 ▶); cell refinement: *APEX2*; data reduction: *SAINT* (Bruker, 2005 ▶); program(s) used to solve structure: *SHELXTL* (Sheldrick, 2008 ▶); program(s) used to refine structure: *SHELXTL*; molecular graphics: *SHELXTL*; software used to prepare material for publication: *SHELXTL* and *PLATON* (Spek, 2003 ▶).

## Supplementary Material

Crystal structure: contains datablocks global, I. DOI: 10.1107/S1600536808037148/tk2323sup1.cif

Structure factors: contains datablocks I. DOI: 10.1107/S1600536808037148/tk2323Isup2.hkl

Additional supplementary materials:  crystallographic information; 3D view; checkCIF report

## Figures and Tables

**Table 1:** Hydrogen-bond geometry (Å, °)

*D*—H⋯*A*	*D*—H	H⋯*A*	*D*⋯*A*	*D*—H⋯*A*
N3—H1*N*3⋯S1^i^	0.891 (15)	2.613 (15)	3.4910 (7)	168.8 (12)
N4—H1*N*4⋯N2	0.885 (14)	2.193 (14)	2.6140 (10)	108.7 (11)
C9—H9*B*⋯S1	0.96	2.78	3.1225 (9)	102

Symmetry code: (i) 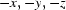.

**Fig. 1.**
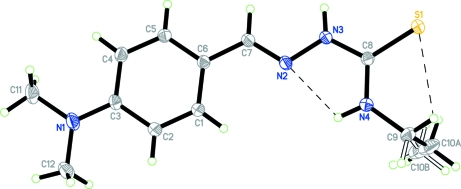
The molecular structure of (I), showing 50% probability displacement ellipsoids and the atomic numbering. The intramolecular contacts are shown as dashed lines.

**Fig. 2.**
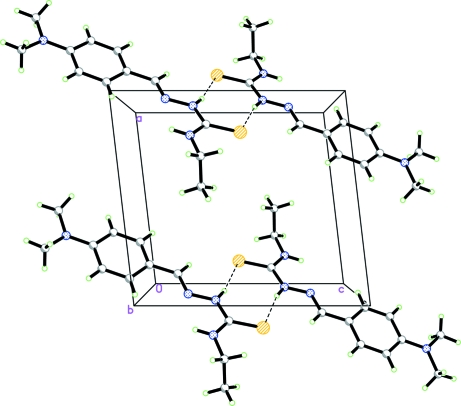
Unit cell contents for the major component of (I), viewed down the b-axis showing dimers linked by R22(8) ring motifs. Intermolecular hydrogen bonds are shown as dashed lines.

## supplementary crystallographic information

### Comment

Thiosemicarbazones have been a subject of extensive investigation (Kayed *et al.*, 2008) because of their ability to coordinate metal ions and their wide range of chemical and biological activities (Valdes-Martinez *et al.*, 1990 & Beraldo *et al.*, 2001). We present herein the X-ray structure of the title thiosemicarbazone derivative, (I).

Compound (I), Fig. I, displays bond lengths and angles comparable to those in related structures (Beraldo *et al.*, 2001; Kayed *et al.*, 2008; Valdes-Martinez *et al.*, 1990). Intramolecular N—H···N and C—H···S contacts generate *S(5)* ring motifs (Bernstein *et al.* 1995), Table 1. The dihedral angle between the phenyl ring and the thiourea unit is 6.30 (6)° which indicates the molecule is almost planar. Intermolecular N—H···S hydrogen bonds generate *R_2_^2^(8)* ring motifs to link molecules into dimers, Fig. 2. The methyl group of the *N*-ethyl residue is disordered and was refined with site occupancies of 0.521 (5)/0.479 (5).

### Experimental

To 4-ethyl-3-thiosemicarbazide (ca. 0.119 g, 1 mmol) dissolved in ethanol (10 ml) was added dropwise an ethanol solution (5 ml) of 4-dimethylaminobenzaldehyde (0.149 g, 1 mmol) with continuous stirring. The solution was heated on a water bath for few minutes until the solution became clear. After cooling to room temperature, yellow crystals of (I) appeared.

### Refinement

The N-bound H atoms were located from a difference Fourier map and refined freely. The remaining H were placed in their calculated positions in the riding model approximation with C–H 0.93–0.96 Å, and with *U*(H) set to 1.2–1.5 times *U*_eq_(C). The presence of large peaks in the difference Fourier map near to the methyl group of (I) was suspected to be due to positional disorder of this fragment. The refined ratio of the site occupancy factors for the disorder parts were calculated to be 0.521 (5)/0.479 (5).

### Crystal data

**Table:**

C_12_H_18_N_4_S	*F*_000_ = 536
*M**_r_* = 250.36	*D*_x_ = 1.231 Mg m^−^^3^
Monoclinic, *P*2_1_/*c*	Mo *K*α radiation λ = 0.71073 Å
Hall symbol: -P 2ybc	Cell parameters from 9949 reflections
*a* = 9.3687 (2) Å	θ = 2.5–39.5º
*b* = 14.1872 (3) Å	µ = 0.23 mm^−^^1^
*c* = 10.2318 (2) Å	*T* = 100 (1) K
β = 96.699 (1)º	Block, yellow
*V* = 1350.68 (5) Å^3^	0.59 × 0.36 × 0.22 mm
*Z* = 4	

### Data collection

**Table:**

Bruker SMART APEXII CCD area-detector diffractometer	6825 independent reflections
Radiation source: fine-focus sealed tube	5566 reflections with *I* > 2σ(*I*)
Monochromator: graphite	*R*_int_ = 0.036
*T* = 100(1) K	θ_max_ = 37.0º
φ and ω scans	θ_min_ = 2.2º
Absorption correction: multi-scan(*SADABS*; Bruker, 2005)	*h* = −15→13
*T*_min_ = 0.879, *T*_max_ = 0.953	*k* = −24→20
30229 measured reflections	*l* = −15→17

### Refinement

**Table:**

Refinement on *F*^2^	Secondary atom site location: difference Fourier map
Least-squares matrix: full	Hydrogen site location: inferred from neighbouring sites
*R*[*F*^2^ > 2σ(*F*^2^)] = 0.041	H atoms treated by a mixture of independent and constrained refinement
*wR*(*F*^2^) = 0.119	*w* = 1/[σ^2^(*F*_o_^2^) + (0.0651*P*)^2^ + 0.2126*P*] where *P* = (*F*_o_^2^ + 2*F*_c_^2^)/3
*S* = 1.04	(Δ/σ)_max_ = 0.001
6825 reflections	Δρ_max_ = 0.74 e Å^−^^3^
176 parameters	Δρ_min_ = −0.39 e Å^−^^3^
Primary atom site location: structure-invariant direct methods	Extinction correction: none

### Special details

**Table:**

Experimental. The low-temperature data was collected with the Oxford Cyrosystem Cobra low-temperature attachment.
Geometry. All e.s.d.'s (except the e.s.d. in the dihedral angle between two l.s. planes) are estimated using the full covariance matrix. The cell e.s.d.'s are taken into account individually in the estimation of e.s.d.'s in distances, angles and torsion angles; correlations between e.s.d.'s in cell parameters are only used when they are defined by crystal symmetry. An approximate (isotropic) treatment of cell e.s.d.'s is used for estimating e.s.d.'s involving l.s. planes.
Refinement. Refinement of *F*^2^ against ALL reflections. The weighted *R*-factor *wR* and goodness of fit *S* are based on *F*^2^, conventional *R*-factors *R* are based on *F*, with *F* set to zero for negative *F*^2^. The threshold expression of *F*^2^ > σ(*F*^2^) is used only for calculating *R*-factors(gt) *etc*. and is not relevant to the choice of reflections for refinement. *R*-factors based on *F*^2^ are statistically about twice as large as those based on *F*, and *R*- factors based on ALL data will be even larger.

### Fractional atomic coordinates and isotropic or equivalent isotropic displacement parameters (Å^2^)

**Table:**

	*x*	*y*	*z*	*U*_iso_*/*U*_eq_	Occ. (<1)
S1	0.16244 (2)	0.114147 (15)	−0.039117 (18)	0.01984 (6)	
N1	−0.30894 (9)	0.10562 (6)	0.82869 (8)	0.02457 (15)	
N2	−0.01025 (7)	0.10572 (5)	0.29168 (6)	0.01690 (12)	
N3	0.02454 (7)	0.08428 (5)	0.16770 (6)	0.01731 (12)	
N4	0.18487 (7)	0.20579 (5)	0.19197 (7)	0.01837 (12)	
C1	−0.09174 (8)	0.13272 (6)	0.55310 (7)	0.01760 (13)	
H1A	−0.0138	0.1680	0.5323	0.021*	
C2	−0.14161 (8)	0.14561 (6)	0.67363 (7)	0.01834 (13)	
H2A	−0.0977	0.1900	0.7319	0.022*	
C3	−0.25861 (8)	0.09230 (6)	0.70986 (7)	0.01732 (13)	
C4	−0.32165 (9)	0.02550 (6)	0.61854 (8)	0.01997 (14)	
H4A	−0.3979	−0.0113	0.6396	0.024*	
C5	−0.27073 (9)	0.01429 (6)	0.49756 (8)	0.01935 (14)	
H5A	−0.3142	−0.0300	0.4388	0.023*	
C6	−0.15624 (8)	0.06743 (5)	0.46132 (7)	0.01601 (12)	
C7	−0.10836 (8)	0.05380 (6)	0.33282 (7)	0.01745 (13)	
H7A	−0.1498	0.0062	0.2786	0.021*	
C8	0.12430 (8)	0.13664 (6)	0.11598 (7)	0.01551 (12)	
C9	0.29288 (10)	0.27026 (6)	0.15428 (9)	0.02484 (16)	
H9A	0.2867	0.3279	0.2024	0.030*	0.521 (5)
H9B	0.2702	0.2848	0.0626	0.030*	0.521 (5)
H9C	0.2877	0.3291	0.1999	0.030*	0.479 (5)
H9D	0.2767	0.2823	0.0614	0.030*	0.479 (5)
C10A	0.4427 (11)	0.2356 (8)	0.1674 (8)	0.0361 (12)	0.521 (5)
H10A	0.5045	0.2841	0.1403	0.054*	0.521 (5)
H10B	0.4486	0.1810	0.1128	0.054*	0.521 (5)
H10C	0.4724	0.2192	0.2575	0.054*	0.521 (5)
C10B	0.4457 (14)	0.2268 (10)	0.1992 (9)	0.0394 (15)	0.479 (5)
H10D	0.4558	0.2160	0.2925	0.059*	0.479 (5)
H10E	0.5188	0.2698	0.1782	0.059*	0.479 (5)
H10F	0.4554	0.1681	0.1544	0.059*	0.479 (5)
C11	−0.43497 (11)	0.05588 (7)	0.86103 (10)	0.02942 (19)	
H11A	−0.5107	0.0619	0.7898	0.044*	
H11B	−0.4657	0.0823	0.9395	0.044*	
H11C	−0.4121	−0.0096	0.8753	0.044*	
C12	−0.24779 (10)	0.17816 (8)	0.91858 (9)	0.02932 (19)	
H12A	−0.1452	0.1710	0.9325	0.044*	
H12B	−0.2864	0.1722	1.0011	0.044*	
H12C	−0.2713	0.2392	0.8815	0.044*	
H1N4	0.1570 (14)	0.2111 (10)	0.2711 (14)	0.032 (3)*	
H1N3	−0.0171 (15)	0.0353 (11)	0.1243 (14)	0.035 (4)*	

### Atomic displacement parameters (Å^2^)

**Table:**

	*U*^11^	*U*^22^	*U*^33^	*U*^12^	*U*^13^	*U*^23^
S1	0.02294 (10)	0.02210 (11)	0.01529 (9)	−0.00401 (7)	0.00569 (6)	−0.00261 (6)
N1	0.0315 (4)	0.0243 (4)	0.0202 (3)	−0.0016 (3)	0.0121 (3)	−0.0011 (2)
N2	0.0198 (3)	0.0176 (3)	0.0137 (2)	−0.0005 (2)	0.0038 (2)	−0.0006 (2)
N3	0.0206 (3)	0.0178 (3)	0.0141 (2)	−0.0036 (2)	0.0043 (2)	−0.0018 (2)
N4	0.0212 (3)	0.0178 (3)	0.0166 (3)	−0.0039 (2)	0.0044 (2)	−0.0022 (2)
C1	0.0179 (3)	0.0185 (3)	0.0166 (3)	−0.0026 (2)	0.0028 (2)	−0.0005 (2)
C2	0.0199 (3)	0.0199 (3)	0.0152 (3)	−0.0015 (3)	0.0024 (2)	−0.0015 (2)
C3	0.0205 (3)	0.0157 (3)	0.0166 (3)	0.0026 (2)	0.0057 (2)	0.0018 (2)
C4	0.0215 (3)	0.0171 (3)	0.0226 (3)	−0.0025 (3)	0.0082 (3)	−0.0007 (3)
C5	0.0213 (3)	0.0167 (3)	0.0208 (3)	−0.0040 (2)	0.0060 (2)	−0.0031 (2)
C6	0.0181 (3)	0.0149 (3)	0.0153 (3)	−0.0008 (2)	0.0032 (2)	−0.0001 (2)
C7	0.0199 (3)	0.0168 (3)	0.0160 (3)	−0.0013 (2)	0.0034 (2)	−0.0013 (2)
C8	0.0162 (3)	0.0156 (3)	0.0148 (3)	0.0000 (2)	0.0017 (2)	0.0010 (2)
C9	0.0294 (4)	0.0199 (4)	0.0269 (4)	−0.0092 (3)	0.0107 (3)	−0.0057 (3)
C10A	0.0211 (12)	0.032 (2)	0.057 (3)	−0.0087 (13)	0.011 (2)	−0.015 (2)
C10B	0.0265 (14)	0.036 (2)	0.057 (4)	−0.0113 (14)	0.009 (3)	−0.011 (3)
C11	0.0374 (5)	0.0252 (4)	0.0291 (4)	−0.0005 (4)	0.0190 (4)	0.0033 (3)
C12	0.0275 (4)	0.0409 (6)	0.0199 (3)	0.0028 (4)	0.0037 (3)	−0.0083 (3)

### Geometric parameters (Å, °)

**Table:**

S1—C8	1.6971 (7)	C6—C7	1.4508 (10)
N1—C3	1.3675 (10)	C7—H7A	0.9300
N1—C11	1.4466 (12)	C9—C10A	1.479 (10)
N1—C12	1.4528 (13)	C9—C10B	1.577 (13)
N2—C7	1.2864 (10)	C9—H9A	0.9600
N2—N3	1.3800 (9)	C9—H9B	0.9600
N3—C8	1.3494 (10)	C9—H9C	0.9600
N3—H1N3	0.890 (15)	C9—H9D	0.9600
N4—C8	1.3365 (10)	C10A—H10A	0.9600
N4—C9	1.4492 (11)	C10A—H10B	0.9600
N4—H1N4	0.883 (14)	C10A—H10C	0.9600
C1—C2	1.3810 (10)	C10B—H10D	0.9600
C1—C6	1.4045 (11)	C10B—H10E	0.9600
C1—H1A	0.9300	C10B—H10F	0.9600
C2—C3	1.4162 (11)	C11—H11A	0.9600
C2—H2A	0.9300	C11—H11B	0.9600
C3—C4	1.4111 (12)	C11—H11C	0.9600
C4—C5	1.3867 (11)	C12—H12A	0.9600
C4—H4A	0.9300	C12—H12B	0.9600
C5—C6	1.3961 (11)	C12—H12C	0.9600
C5—H5A	0.9300		
			
C3—N1—C11	120.75 (8)	C10A—C9—H9A	110.5
C3—N1—C12	120.62 (8)	C10B—C9—H9A	106.9
C11—N1—C12	118.22 (7)	N4—C9—H9B	108.3
C7—N2—N3	115.40 (7)	C10A—C9—H9B	105.1
C8—N3—N2	119.20 (7)	C10B—C9—H9B	117.3
C8—N3—H1N3	121.2 (9)	H9A—C9—H9B	107.4
N2—N3—H1N3	119.6 (9)	N4—C9—H9C	110.0
C8—N4—C9	124.80 (7)	C10A—C9—H9C	110.3
C8—N4—H1N4	116.4 (9)	C10B—C9—H9C	107.1
C9—N4—H1N4	118.7 (9)	H9B—C9—H9C	105.7
C2—C1—C6	121.36 (7)	N4—C9—H9D	110.0
C2—C1—H1A	119.3	C10A—C9—H9D	100.8
C6—C1—H1A	119.3	C10B—C9—H9D	113.0
C1—C2—C3	121.05 (7)	H9A—C9—H9D	110.1
C1—C2—H2A	119.5	H9C—C9—H9D	108.4
C3—C2—H2A	119.5	C9—C10A—H10A	109.5
N1—C3—C4	121.35 (7)	C9—C10A—H10B	109.5
N1—C3—C2	121.11 (7)	C9—C10A—H10C	109.5
C4—C3—C2	117.53 (7)	C9—C10B—H10D	109.5
C5—C4—C3	120.47 (7)	C9—C10B—H10E	109.5
C5—C4—H4A	119.8	H10D—C10B—H10E	109.5
C3—C4—H4A	119.8	C9—C10B—H10F	109.5
C4—C5—C6	122.02 (7)	H10D—C10B—H10F	109.5
C4—C5—H5A	119.0	H10E—C10B—H10F	109.5
C6—C5—H5A	119.0	N1—C11—H11A	109.5
C5—C6—C1	117.54 (7)	N1—C11—H11B	109.5
C5—C6—C7	119.81 (7)	H11A—C11—H11B	109.5
C1—C6—C7	122.65 (7)	N1—C11—H11C	109.5
N2—C7—C6	121.90 (7)	H11A—C11—H11C	109.5
N2—C7—H7A	119.1	H11B—C11—H11C	109.5
C6—C7—H7A	119.1	N1—C12—H12A	109.5
N4—C8—N3	116.26 (6)	N1—C12—H12B	109.5
N4—C8—S1	124.10 (6)	H12A—C12—H12B	109.5
N3—C8—S1	119.62 (6)	N1—C12—H12C	109.5
N4—C9—C10A	116.8 (4)	H12A—C12—H12C	109.5
N4—C9—C10B	108.3 (4)	H12B—C12—H12C	109.5
N4—C9—H9A	108.4		
			
C7—N2—N3—C8	178.60 (7)	C4—C5—C6—C7	−179.19 (8)
C6—C1—C2—C3	0.93 (12)	C2—C1—C6—C5	−1.47 (12)
C11—N1—C3—C4	−4.17 (13)	C2—C1—C6—C7	178.59 (8)
C12—N1—C3—C4	−176.68 (8)	N3—N2—C7—C6	179.78 (7)
C11—N1—C3—C2	175.45 (8)	C5—C6—C7—N2	174.99 (8)
C12—N1—C3—C2	2.93 (13)	C1—C6—C7—N2	−5.08 (12)
C1—C2—C3—N1	−179.39 (8)	C9—N4—C8—N3	−179.39 (8)
C1—C2—C3—C4	0.24 (12)	C9—N4—C8—S1	−1.06 (12)
N1—C3—C4—C5	178.80 (8)	N2—N3—C8—N4	1.21 (11)
C2—C3—C4—C5	−0.82 (12)	N2—N3—C8—S1	−177.20 (5)
C3—C4—C5—C6	0.26 (13)	C8—N4—C9—C10A	−81.0 (4)
C4—C5—C6—C1	0.88 (12)	C8—N4—C9—C10B	−90.9 (4)

### Hydrogen-bond geometry (Å, °)

**Table:**

*D*—H···*A*	*D*—H	H···*A*	*D*···*A*	*D*—H···*A*
N3—H1N3···S1^i^	0.891 (15)	2.613 (15)	3.4910 (7)	168.8 (12)
N4—H1N4···N2	0.885 (14)	2.193 (14)	2.6140 (10)	108.7 (11)
C9—H9B···S1	0.96	2.78	3.1225 (9)	102

Symmetry codes: (i) −*x*, −*y*, −*z*.
